# Phenotypic Overlap of Roberts and Baller-Gerold Syndromes in Two Patients With Craniosynostosis, Limb Reductions, and *ESCO2* Mutations

**DOI:** 10.3389/fped.2019.00210

**Published:** 2019-05-28

**Authors:** Elisa Adele Colombo, Hatice Mutlu-Albayrak, Yousef Shafeghati, Mine Balasar, Juliette Piard, Davide Gentilini, Anna Maria Di Blasio, Cristina Gervasini, Lionel Van Maldergem, Lidia Larizza

**Affiliations:** ^1^Genetica Medica, Dipartimento di Scienze della Salute, Università degli Studi di Milano, Milan, Italy; ^2^Department of Pediatric Genetics, Cengiz Gökcek Maternity and Children's Hospital, Gaziantep, Turkey; ^3^Sarem Cell Research Center and Medical Genetics Department, Sarem Women Hospital, Tehran, Iran; ^4^Department of Medical Genetics, Meram Medical Faculty, Necmettin Erbakan University, Konya, Turkey; ^5^Centre de génétique humaine CHU, Université de Franche-Comté, Besançon, France; ^6^Laboratorio di Biologia Molecolare, Istituto Auxologico Italiano, IRCCS, Milan, Italy; ^7^Department of Brain and Behavioral Sciences, University of Pavia, Pavia, Italy; ^8^Laboratorio di Citogenetica e Genetica Molecolare Umana, Istituto Auxologico Italiano, IRCCS, Milan, Italy

**Keywords:** Roberts syndrome, Baller-Gerold syndrome, *RECQL4*, *ESCO2*, patient management, genetic counseling, differential diagnosis

## Abstract

Baller-Gerold (BGS, MIM#218600) and Roberts (RBS, MIM#268300) syndromes are rare autosomal recessive disorders caused, respectively, by biallelic alterations in *RECQL4* (MIM^*^603780) and *ESCO2* (MIM^*^609353) genes. Common features are severe growth retardation, limbs shortening and craniofacial abnormalities which may include craniosynostosis. We aimed at unveiling the genetic lesions underpinning the phenotype of two unrelated children with a presumptive BGS diagnosis: patient 1 is a Turkish girl with short stature, microcephaly, craniosynostosis, seizures, intellectual disability, midface hemangioma, bilateral radial and thumb aplasia, tibial hypoplasia, and pes equinovarus. Patient 2 is an Iranian girl born to consanguineous parents with craniosynostosis, micrognathism, bilateral radial aplasia, thumbs, and foot deformity in the context of developmental delay. Upon negative *RECQL4* test, whole exome sequencing (WES) analysis performed on the two trios led to the identification of two different *ESCO2* homozygous inactivating variants: a previously described c.1131+1G>A transition in patient 1 and an unreported deletion, c.417del, in patient 2, thus turning the diagnosis into Roberts syndrome. The occurrence of a Baller-Gerold phenotype in two unrelated patients that were ultimately diagnosed with RBS demonstrates the strength of WES in redefining the nosological landscape of rare congenital malformation syndromes, a premise to yield optimized patients management and family counseling.

## Background

Baller-Gerold syndrome (BGS, OMIM#218600) (1) and Roberts syndrome (RBS, OMIM#268300) (2) are two rare autosomal recessive congenital disorders with clinical overlap and a broad phenotypic variability.

The major clinical findings of BGS involve the skeleton: all patients display craniosynostosis of any or all cranial sutures and pre-axial upper limb defects, mainly hypoplasia/aplasia of radii, ulnae, and/or thumbs and malformation or absence of some carpal and metacarpal bones. The spectrum of BGS manifestations includes also growth delay, anomalies of lower limbs (absent patellae, genu valgum, club feet), imperforate or anteriorly placed anus, rectovaginal fistula, renal and cardiac (tetralogy of Fallot, ventricular septal defects) malformations, skin alterations (poikiloderma, hyper- and hypopigmentation, hemangioma, nevus flammeus), facial dysmorphisms (telecanthus, malpositioned ears, prominent/depressed nasal bridge, high-arched/cleft palate, micrognathia), and occasionally intellectual disability. Many BGS patients die within the first year of life and a few may develop malignancies early in life (3, 4).

About 70 clinically diagnosed BGS patients are reported in the literature but, subsequently to further in-depth analysis, a different diagnosis has been formulated for almost 20 of them (3, 5–9). To date, biallelic alterations in *RECQL4* gene (OMIM^*^603780), encoding a protein of the RECQ helicase family with multiple functions in the maintenance of genome integrity, have been found in a subgroup of 11 BGS patients from 7 families (1, 4, 10–12). To note, 4 out of 5 BGS patients, who could be evaluated, present with poikiloderma, a chronic cutaneous alteration manifesting in the first years of life in most of the *RECQL4*-mutated patients (4, 10, 11).

Roberts syndrome, which includes SC Phocomelia syndrome (OMIM#269000), is a multiple malformation syndrome (2) described in at least 230 patients belonging to 167 different families. Its main clinical features include severe pre- and post-natal growth retardation, symmetrical tetra-limb reduction of various degree (usually more severe in upper limbs) associated to skeletal defects including absence or reduction in length of humeri, radii and thumbs, ulna deformity, oligodactyly, and neurological signs, such as mild to severe intellectual disability and seizures. Craniofacial dysmorphisms (hypertelorism, cleft lip, cleft palate, nose and ears anomalies), microcephaly, facial hemangioma, congenital heart defects, polycystic or dysplastic kidneys, and enlarged genitalia are frequently observed in RBS patients. The most severe phenotypes usually lead to fetal death or in the first months of life. SC Phocomelia is considered the mild clinical variant of RBS (13) and is characterized by mild symmetric limb reductions, joint contractures, microcephaly, midfacial hemangioma, cloudy corneas, and mild or borderline intellectual disability. Survival to adulthood is common (14).

At the cellular level, the chromosomal instability hallmark of premature centromere separation and splitting of the heterochromatic regions, also termed “heterochromatic repulsion” (HR), characterize metaphase spreads of RBS-SC Phocomelia cells (15).

Biallelic pathogenic variants in *ESCO2* (*establishment of cohesion 1 homolog 2*; OMIM^*^609353) gene located at 8p21.1, have been identified in RBS and SC Phocomelia (2). *ESCO2* encodes an acetyltransferase of the cohesion establishing complex involved in sister chromatids cohesion during DNA replication and double-strand breaks repair (16). No genotype-phenotype correlation has been established so far. A variable clinical presentation between sibs, including both RBS and SC Phocomelia phenotypes, suggests that modifier genes and epigenetic factors contribute to this uncommon variability among autosomal recessive disorders (2). Mildly affected children are probably overrepresented in the published clinical descriptions since they are more likely to survive and to come to medical attention.

We herein report on two unrelated cases of RBS who were first clinically diagnosed as BGS and, upon negative *RECQL4* test, were found by whole exome sequencing (WES) to harbor homozygous mutations in *ESCO2* gene. One of the observed variants, yet unreported, expands *ESCO2* mutational spectrum.

## Case Presentation

We describe two affected patients of two unrelated families (1 and 2) with a syndromic phenotype suggestive of BGS referred to our laboratory by clinical geneticists and pediatricians. Appropriate written informed consent to genetic analysis and authorization to photos collection were obtained from the parents of the patients for the publication of the case report and any potentially-identifying information/images. The study protocol was approved by the Research Ethics Board of Istituto Auxologico Italiano, Milan, Italy.

### Clinical Report of Family 1

**Figure 1:**
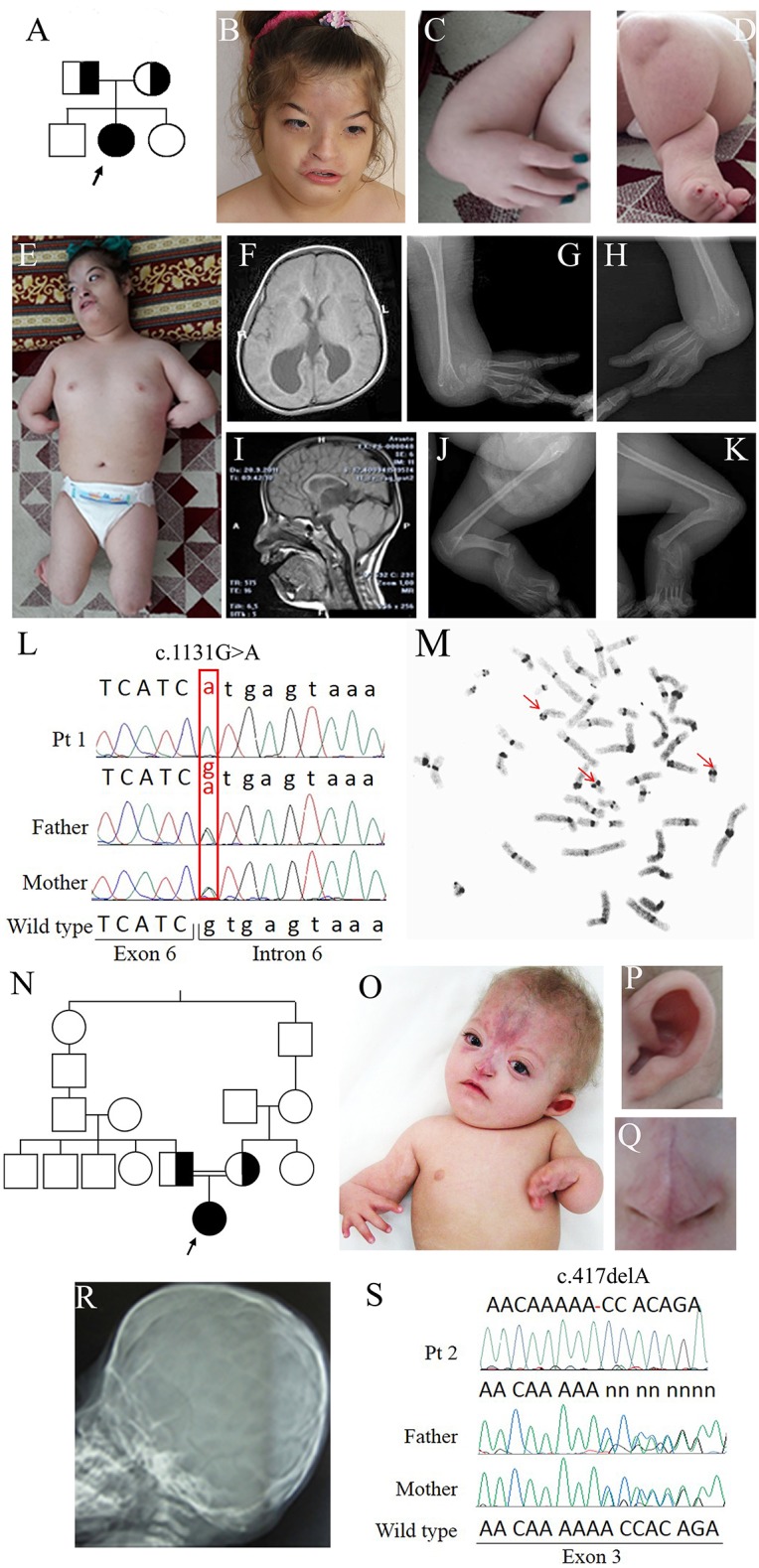
Clinical and molecular features of patients 1 and 2. **(A)** Pedigree of family 1: the arrow indicates the proband. **(B)** Face of patient 1 at 12 years showing arched eyebrows, telecanthus, epicanthal folds, hemangioma on the frontal region, small and flared nose, short philtrum, and a wide mouth with downturned corners. **(C)** Forearm aplasia, manus varus deformity, and thumb aplasia. **(D)** Close-up of the left leg showing short cruris, flexion deformity on knees, and pes equinovarus. **(E)** Overall photo of patient 1 showing dysmorphisms of the face and malformations of arms and legs. **(F)** Coronal cross-sectional areas obtained with cranial MR T2 showing asymmetric colpocephalic expansion of lateral ventricles occipital horns. **(G,H)** X-rays images of arms showing bilaterally aplasia of radius, ulna and thumb, hypoplasia of middle phalanx, fusion of 4–5 metacarpals and carpal bones. **(I)** Sagittal cross-sectional areas obtained with cranial MR T2 showing partial corpus callosum agenesis. **(J,K)** X-rays images of the legs evidencing bilaterally fibular aplasia, femoral-tibial synostosis, fusion of tarsal bones, short and bowed tibias. **(L)** Chromatograms of *ESCO2* sequence around c.1131+1G>A in intron 6 of the affected girl (top) and her obligate carriers parents (middle and bottom). Fex6 (5′-gaggaccaggatttgagtgtt-3′) and Rex6 (5′-accacctacaactcccattct-3′) primers were used to amplify this region. **(M)** C-banded metaphase spread showing premature centromere separation with puffing at the centromere and heterochromatic regions (arrowed). **(N)** Pedigree of family 2 with RBS-affected patient arrowed. **(O)** Photograph of patient 2, showing forearm aplasia, varus deformity of hands, thumb aplasia, extended capillary malformation, and sparse hair. Magnifications showing the protruding cupped ear **(P)** and the short nose with underdeveloped alae nasi and narrow and sharp nasal ridge **(Q)**. **(R)** Coronal craniosynostosis and brachycephaly profile view of head X-rays. **(S)** Chromatograms of *ESCO2* sequence around c.417del alteration in exon 3 in the affected girl (top) and her obligate carriers parents. The exon 3-specific amplicons were obtained using primers Fex3 (5′-gcaaatcaaggctcacca-3′) and Rex3 (5′-ttttggctcagaacccga-3′).

Proband 1 is the second child of Turkish parents originating from the same geographical area. She was born in 2005 at full-term after an uneventful pregnancy and presented with mesomelic shortening of limbs and bilateral thumb aplasia. She has an elder brother who suffered from epilepsy, motor delay and intellectual disability, and a 1-year-old sister who had an epileptic attack at 8 months of age (Figure 1A).

Early in infancy, bilateral corneal clouding was noted and surgically removed. Due to eye proptosis and cranial deformity, she underwent a skull radiograph and CT scan that indicated sagittal and coronal premature fusion. She manifested motor and developmental delay. Language was delayed and limited to a few words without the ability to construct sentences.

Generalized seizures, responsive to levetiracetam, occurred when she was 8-year old; her height was at −8.5 SD (80 cm), weight at −4.8 SD (10 kg), and OFC at −7 SD (43 cm).

On clinical examination at 12 years, she could not follow an object with her eyes nor speak or respond to questions. Facial dysmorphisms included low anterior and posterior hairlines, arched eyebrows, telecanthus, epicanthal folds, eye proptosis, left exotropia, cutaneous hemangioma on the frontal region expanding to the nose which is small and flared, a short philtrum, wide mouth with downturned corners and high-arched palate (Figure 1B). Her upper limbs were extremely short, thumbs absent while fingers were long and tibias short and bowed (Figures 1C,E). There was an equinovarus deformity of the right foot and calcaneus valgus deformity on the left foot, overriding toes and major joints contractures (Figures 1D,E). The skeletal survey showed bilateral aplasia of radius, ulna, and thumb (Figures 1G,H,J,K) while brain MRI evidenced asymmetric dilatation of lateral ventricles and agenesis of corpus callosum (Figures 1F,I). Karyotype was 46,XX on peripheral lymphocytes. Array-CGH was normal.

### Clinical Report of Family 2

Proband 2 is a 2-year-old Iranian girl born to consanguineous parents, presenting with a malformation syndrome comprising craniosynostosis, bilateral radial ray hypoplasia and absent thumbs (Figures 1N,O,R). The pregnancy was unremarkable till 6 months of gestation when ultrasonographic examination revealed oligohydramnios and bilateral club feet. The baby was born at 29 weeks of gestation and her birth weight was 720 g. A large frontal hemangioma and craniofacial dysmorphisms including large alae nasi, small nose with deep nasal bridge, arched palate, micrognathia, simple ears, and short neck were observed since birth (Figures 1O–Q). She had bilateral hypoplastic radius, oligodactyly, and knee joints stiffness. Bilateral glaucoma and hypotonia were also noted. Neurodevelopmental delay and hypothyroidism were recorded.

## Laboratory Investigations

A provisional clinical diagnosis of Baller-Gerold syndrome was made for both patients based on the association of craniosynostosis and radial ray defects. However, *RECQL4* Sanger sequencing carried on as previously described (17) didn't evidence any significant sequence alteration.

For both families, WES was performed on 50 ng of genomic DNA of each member of the trio (index patient and parents) prepared using the Illumina® Nextera® Rapid Capture Exome kit (Illumina) and sequenced on Illumina HiSeq2500 sequencer (Illumina). Reads were aligned against the human reference genome (hg 19/GRCh37) using the Burrows-Wheeler Alignment tool (18). The variant calling was performed with the SAM tool (19) and the variant annotation with GATK (20).

Given the rarity of the suspected disease, the variant list was filtered according to an allele frequency ≤0.1% according to the ExAC Browser of Broad Institute (21) and 1,000 Genomes (22) databases. Subsequent filtering steps sorted out variants following the autosomal recessive inheritance model as well as their functional impact (i.e., non-sense, affecting the canonical splice-site regions or non-synonymous) and the prediction of Polyphen (23) and SIFT (24) bioinformatics tools.

Filtering about 43,500 variants in each sample led to the identification of two different *ESCO2* (NG_008117.1) homozygous alterations in the probands: c.1131+1G>A in intron 6 of patient 1 and c.417del in exon 3 of patient 2 [sequence variants were termed according to HGVS recommendations (25)]. Sanger sequencing confirmed the biallelic pathogenic alterations in the probands and the carrier status of their parents (Figures 1L,S) and of the elder brother of patient 1. The splice donor variant c.1131+1G>A has a MAF <0.01 and has been previously reported (13, 26–28) while the c.417del is absent from public databases, has not been described to date and has been submitted to LOVD database (https://databases.lovd.nl/shared/genes/ESCO2).

The final diagnosis of Roberts syndrome was done in both cases. Moreover, C-banding chromosome analysis of patient 1 showed the premature centromere separation and heterochromatin repulsion known to be pathognomonic for RBS (Figure 1M).

## Discussion

**Figure 2:**
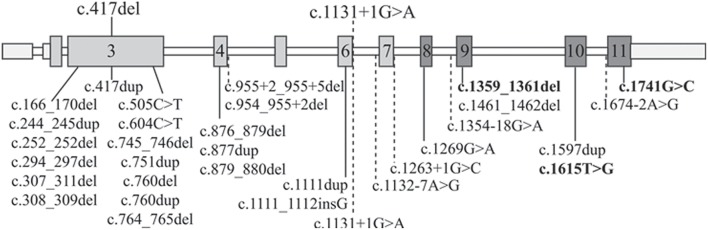
*ESCO2* pathogenic alterations. Schematic diagram of full-length *ESCO2* gene: exons are depicted as boxes while introns as thin bars. Exons encoding the acetyltransferase domain are in dark gray. The two mutations carried by the patients herein described are over the gene schematic while all the 32 known pathogenic alterations found in RBS-SC Phocomelia patients are listed below. The only 3 “non-inactivating” mutations (2 missense mutations and a three-nucleotide deletion) are bolded. The horizontal dashed lines indicate intronic mutations leading to miss-splicing.

To better compare the characteristics of our patients with those of the RBS-SC Phocomelia patients reported in the literature with the same recurrent mutation of case 1 or with a different mutation but affecting the same nucleotide of case 2, we provide an overview of all known *ESCO2* pathogenic variants. As can be seen at glance in Figure 2 there is no hot spot as the mutations are scattered throughout the gene. To date, out of 33 different pathogenic variants described in 49 families, 31 are inactivating mutations leading to protein truncation and lack of the evolutionarily conserved C-terminal domain where the acetyltransferase activity is harbored.

The c.1131+1G>A alteration of family 1 has been previously reported in literature both in homozygous (13, 26, 27) and compound heterozygous (28) state and it has been demonstrated that the mutated allele codifies for an aberrant transcript lacking exon 6 (r.1014_1131del118) and exposing a premature stop codon (p.Arg338fs^*^17) (13). The clinical phenotype of the three patients with the c.1131+1G>A homozygous splicing mutation is, although variable, ranging from the mildest phenotype of the adult patient with SC Phocomelia syndrome (13) to the most severe of RBS in an infant prematurely died (27). All patients share dysmorphisms, microcephaly, and skeletal defects. Compound heterozygosity for this splice site alteration and the c.954_955+2del sequence alteration was also described in a female RBS fetus with multiple malformations (limbs alterations, hygroma, and intrauterine growth retardation) deceased at 18 weeks of gestation (28). At difference from our patient, none of the described patients was reported to suffer from epilepsy as well as craniosynostosis, which are rarely described in RBS patients (13, 15, 29, 30). However, one may suppose that epilepsy in our RBS patient may represent a comorbid condition due to segregation of a common recessive epilepsy gene from the likely related parents, given that also proband's siblings suffer from seizures but do not have RBS.

As regards the single nucleotide c.417del deletion (Figure 2) identified in patient 2, it has not been reported in the literature so far. It is worth noting that a duplication involving the same nucleotide has been observed (31) in a Turkish fetus with RBS (29).

Our RBS patients masqueraded as Baller-Gerold syndrome patients due to craniosynostosis, uncommon in RBS, and radial ray hypoplasia, a major BGS sign which in RBS presents in the general context of upper limb reductions. In addition, both patients had a severe developmental delay, midface hemangioma, striking facial dysmorphism more severe than that usually observed in coronal or sagittal craniosynostosis. Overall, these clinical signs and symptoms encompass and surpass the classical Baller-Gerold clinical picture.

Table 1 summarizes the distinctive clinical features of BGS and RBS individuals whose clinical phenotype could be confirmed by molecular analysis of the respective *RECQL4* and *ESCO2* genes. Craniosynostosis is a distinctive sign of BGS as it has been reported in 82% (9/11) of BGS patients with *RECQL4* alterations (4, 10–12, 32, 33) but in RBS with *ESCO2* pathogenic variants only in the 2 patients herein described and in 2 patients of the literature, for whom no details or images are provided (29). Conversely, hemangioma on the face, recorded in 30 *ESCO2*+ patients (29, 34, 35) and in only one *RECQL4*+ patient (10, 32), as well as intellectual disability, assessed in 23 *ESCO2*+ patients (29, 34) and in 1 *RECQL4*+ patient (4), are prominent in RBS. In addition, cognitive impairment is more severe in RBS than BGS: most RBS patients show not only developmental delay but also a severe intellectual disability even if marked variability exists between RBS and SC Phocomelia (2).

**Table 1:** Summary of the major clinical signs of BGS and RBS according to the clinically and molecularly investigated patients with either syndrome.

	**BGS *RECQL4+***	**RBS *ESCO2*+**
N° of patients (n° of fetuses)	11 (5)	61 (13)
Pre/post-natal growth delay	4	52
Craniosynostosis	9	4
Craniofacial dysmorphisms	4	38
Hemangioma	1	30
Upper limbs malformations	11	61
Lower limbs malformations	7	61
Intellectual disability/developmental delay	1	23

Moreover, 90% (10/11) BGS (10–12, 32, 33, 36) and all RBS (14, 27–29, 34, 35, 37, 38) patients present with radial alterations (hypo/aplasia) and most of them manifest additional upper and lower limb malformations. Pre-axial upper limb defects are similar in BGS and RBS: ulnae hypoplasia, club hands, thumbs aplasia/hypoplasia, clinodactyly, and oligodactyly, though humeral reduction can be also present in RBS.

Conversely, marked differences in lower limb malformations exist between BGS and RBS (Table 1): hip and knee joint dislocation, patellar aplasia, or hypoplasia, hypoplasia of great toe, club feet have been reported in BGS while in RBS lower limb malformations include femoral, tibial and fibular hypoplasia or aplasia, club feet, and knee joint dislocation. As known, RBS malformations of upper limbs are more severe than those of lower limbs, and some patients present skeletal defects only in the arms (2).

Several additional anomalies have been observed in BGS and RBS patients with a severe phenotype, who can present multiple additional abnormalities coupling only with one syndrome. Poikiloderma (4, 10, 11, 33), imperforate and/or anteriorly positioned anus (4, 10, 32), feeding difficulties (10, 11, 33), hypospadias and undescended testes (33), and osseous demineralization (11, 33) have been recorded only in BGS patients, while heart defects (14, 27, 29, 34, 35, 38) and enlargement of phallus/clitoris (29) have been reported only in RBS. Before the availability of the molecular test, heart defects as well as seizure, have been included in the spectrum of BGS signs (39) but none of BGS patients with *RECQL4* alterations manifested these clinical findings, a data raising the possibility that many patients described in the past had received the wrong diagnosis of BGS, as it has been confirmed in a few cases (7–9).

The clinical overlap of BGS, a *RECQL4*-associated disease, and RBS cohesinopathy is accounted for by the interconnected pathways of the respective genes, as evidenced by the inclusion of RECQL4 among the accessory proteins acting in the cohesin pathway (40) and by downregulation of *RECQL4* in Cornelia de Lange (OMIM#122470) patients (41). Both RECQL4 and ESCO2 contribute to the maintenance of correct chromosomal segregation and syndrome-specific hallmarks of chromosomal instability are observed when both alleles of these key genes are deranged (42, 43).

Switching back to BGS-RBS phenotypic similarity one has to note that this analysis suffers from the limited number of survived and clinically evaluated BGS individuals (out of 11 cases there are five terminated pregnancies (10, 11, 36) and one death occurred few minutes after birth (10, 32) vs. the much higher number of RBS patients (29 patients >1 year) (14, 29, 34). Such disproportion makes hard to compare the frequency of especially rare clinical findings, such as craniosynostosis, between RBS and BGS patients. A further difficulty arises from the wide clinical expressivity of both syndromes, deserving a consistent number of described patients, not yet available for BGS, to classify signs recorded only in one or few patients.

## Concluding Remarks

The similarity in upper limb malformations and pre/post-natal growth delay in BGS and RBS joined to the peculiarity of additional syndrome-specific malformations, can help clinicians in the clinical diagnosis.

However, the diagnosis of ultra-rare syndromes characterized by a huge clinical spectrum, such as BGS and RBS, is quite challenging and benefits enormously from the application of contemporary molecular genomics techniques allowing the unequivocal identification of the genetic lesion behind the disease. The suspected diagnosis can be confirmed/excluded, hence enhancing optimized patient management/follow-up, family counseling and appropriate therapy provision.

## Ethics Statement

The study protocol was approved by the Research Ethics Board of Istituto Auxologico Italiano, Milano, Italy.

For each patient, appropriate written informed consent to genetic analysis and authorization to photos collection and publication were obtained by parents.

## Author Contributions

EAC carried out the genetic analyses and analyzed WES variants, drafted the manuscript. HMA examined patient 1 and summarized clinical data and critically reviewed the manuscript. YS examined patient 2 and summarized clinical data. MB examined patient 1, summarized clinical data, and performed cytogenetic analysis. JP examined patients 2 and summarized clinical data. DG performed next-generation sequencing. AMDB supported WES analyses. CG supported genetic and variants filtering analyses and reviewed the manuscript. LV examined patients, summarized clinical data, and critically reviewed the manuscript. LL conceptualized and designed the study, coordinated the work, drafted and reviewed the manuscript. All authors read and approved the final manuscript.

### Conflict of Interest Statement

The authors declare that the research was conducted in the absence of any commercial or financial relationships that could be construed as a potential conflict of interest.

## References

[1] Van Maldergem L Baller-Gerold Syndrome. In: Adam MP Ardinger HH Pagon RA Wallace SE Bean LJ Stephens K editors. GeneReviews® Seattle, WA: University of Washington (1993). Available online at: http://www.ncbi.nlm.nih.gov/books/NBK1204/. (accessed April 15, 2019). 20301383

[2] Gordillo M Vega H Jabs EW Roberts Syndrome. In: Adam MP Ardinger HH Pagon RA Wallace SE Bean LJ Stephens K editors. GeneReviews® Seattle, WA: University of Washington (1993). Available online at: http://www.ncbi.nlm.nih.gov/books/NBK1153/. (accessed April 15, 2019).

[3] Preis S Majewski F Körholz D Göbel U. Osteosarcoma in a 16-year-old boy with Baller-Gerold syndrome. Clin Dysmorphol. (1995) 4:161–8. 10.1097/00019605-199504000-00009 7606324

[4] Debeljak M Zver A Jazbec J. A patient with Baller-Gerold syndrome and midline NK/T lymphoma. Am J Med Genet A. (2009) 149A:755–9. 10.1002/ajmg.a.32736 19291770

[5] Farrell SA Paes BA Lewis ME. Fanconi anemia in a child previously diagnosed as Baller-Gerold syndrome. Am J Med Genet. (1994) 50:98–9. 10.1002/ajmg.1320500123 8160763

[6] Rossbach HC Sutcliffe MJ Haag MM Grana NH Rossi AR Barbosa JL. Fanconi anemia in brothers initially diagnosed with VACTERL association with hydrocephalus, and subsequently with Baller-Gerold syndrome. Am J Med Genet. (1996) 61:65–7. 10.1002/(SICI)1096-8628(19960102)61:1<65::AID-AJMG12>3.0.CO;2-U 8741921

[7] Gripp KW Stolle CA Celle L McDonald-McGinn DM Whitaker LA Zackai EH. TWIST gene mutation in a patient with radial aplasia and craniosynostosis: further evidence for heterogeneity of Baller-Gerold syndrome. Am J Med Genet. (1999) 82:170–6. 10.1002/(SICI)1096-8628(19990115)82:2<170::AID-AJMG14>3.0.CO;2-X 9934984

[8] Seto ML Lee SJ Sze RW Cunningham ML. Another TWIST on Baller-Gerold syndrome. Am J Med Genet. (2001) 104:323–30. 10.1002/ajmg.10065 11754069

[9] Piard J Aral B Vabres P Holder-Espinasse M Mégarbané A Gauthier S. Search for ReCQL4 mutations in 39 patients genotyped for suspected Rothmund-Thomson/Baller-Gerold syndromes. Clin Genet. (2015) 87:244–51. 10.1111/cge.12361 24635570

[10] Van Maldergem L Siitonen HA Jalkh N Chouery E De Roy M Delague V. Revisiting the craniosynostosis-radial ray hypoplasia association: Baller-Gerold syndrome caused by mutations in the RECQL4 gene. J Med Genet. (2006) 43:148–52. 10.1136/jmg.2005.031781 15964893 PMC2564634

[11] Siitonen HA Sotkasiira J Biervliet M Benmansour A Capri Y Cormier-Daire V. The mutation spectrum in RECQL4 diseases. Eur J Hum Genet. (2009) 17:151–8. 10.1038/ejhg.2008.154 18716613 PMC2986053

[12] Kaneko H Izumi R Oda H Ohara O Sameshima K Ohnishi H Nationwide survey of Baller-Gerold syndrome in Japanese population. Mol Med Rep. (2017) 15:3222–4. 10.3892/mmr.2017.6408 28358413

[13] Schüle B Oviedo A Johnston K Pai S Francke U. Inactivating mutations in ESCO2 cause SC phocomelia and Roberts syndrome: no phenotype-genotype correlation. Am J Hum Genet. (2005) 77:1117–28. 10.1086/498695 16380922 PMC1285169

[14] Goh ES-Y Li C Horsburgh S Kasai Y Kolomietz E Morel CF. The Roberts syndrome/SC phocomelia spectrum–a case report of an adult with review of the literature. Am J Med Genet A. (2010) 152A:472–8. 10.1002/ajmg.a.33261 20101700

[15] Tomkins D Hunter A Roberts M. Cytogenetic findings in Roberts-SC phocomelia syndrome(s). Am J Med Genet. (1979) 4:17–26. 10.1002/ajmg.1320040104 495649

[16] Unal E Heidinger-Pauli JM Koshland D. DNA double-strand breaks trigger genome-wide sister-chromatid cohesion through Eco1 (Ctf7). Science. (2007) 317:245–8. 10.1126/science.1140637 17626885

[17] Colombo EA Locatelli A Cubells Sánchez L Romeo S Elcioglu NH Maystadt I. Rothmund-Thomson Syndrome: insights from new patients on the genetic variability underpinning clinical presentation and cancer outcome. Int J Mol Sci. (2018) 19:E1103. 10.3390/ijms19041103 29642415 PMC5979380

[18] Li H Durbin R. Fast and accurate long-read alignment with Burrows-Wheeler transform. Bioinformatics. (2010) 26:589–95. 10.1093/bioinformatics/btp698 20080505 PMC2828108

[19] Li H Handsaker B Wysoker A Fennell T Ruan J Homer N. The sequence alignment/map format and SAMtools. Bioinformatics. (2009) 25:2078–9. 10.1093/bioinformatics/btp352 19505943 PMC2723002

[20] DePristo MA Banks E Poplin R Garimella KV Maguire JR Hartl C. A framework for variation discovery and genotyping using next-generation DNA sequencing data. Nat Genet. (2011) 43:491–8. 10.1038/ng.806 21478889 PMC3083463

[21] Lek M Karczewski KJ Minikel EV Samocha KE Banks E Fennell T. Analysis of protein-coding genetic variation in 60,706 humans. Nature. (2016) 536:285–91. 10.1038/nature19057 27535533 PMC5018207

[22] The 1000 Genomes Project Consortium A global reference for human genetic variation. Nature. (2015) 526:68–74. 10.1038/nature15393 26432245 PMC4750478

[23] Adzhubei IA Schmidt S Peshkin L Ramensky VE Gerasimova A Bork P. A method and server for predicting damaging missense mutations. Nat Methods. (2010) 7:248–9. 10.1038/nmeth0410-248 20354512 PMC2855889

[24] Ng PC Henikoff S. SIFT: predicting amino acid changes that affect protein function. Nucleic Acids Res. (2003) 31:3812–4. 10.1093/nar/gkg509 12824425 PMC168916

[25] den Dunnen TJ Dalgleish R Maglott DR Hart RK Greenblatt MS McGowan-Jordan J. HGVS Recommendations for the Description of Sequence Variants: 2016 Update. Hum Mutat. (2016) 37:564–9. 10.1002/humu.22981 26931183

[26] Gordillo M Vega H Trainer AH Hou F Sakai N Luque R. The molecular mechanism underlying Roberts syndrome involves loss of ESCO2 acetyltransferase activity. Hum Mol Genet. (2008) 17:2172–80. 10.1093/hmg/ddn116 18411254

[27] Dogan M Firinci F Balci YI Zeybek S Ozgürler F Erdogan I. The Roberts syndrome: a case report of an infant with valvular aortic stenosis and mutation in ESCO2. J Pak Med Assoc. (2014) 64:457–60. 24864645

[28] Dupont C Bucourt M Guimiot F Kraoua L Smiljkovski D Le Tessier D. 3D-FISH analysis reveals chromatid cohesion defect during interphase in Roberts syndrome. Mol Cytogenet. (2014) 7:59. 10.1186/s13039-014-0059-6 25320640 PMC4197286

[29] Vega H Trainer AH Gordillo M Crosier M Kayserili H Skovby F. Phenotypic variability in 49 cases of ESCO2 mutations, including novel missense and codon deletion in the acetyltransferase domain, correlates with ESCO2 expression and establishes the clinical criteria for Roberts syndrome. J Med Genet. (2010) 47:30–7. 10.1136/jmg.2009.068395 19574259

[30] Eylon S Beeri M Joseph K Meyer S. Femorotibial ankylosis in a child with Roberts syndrome: an ≪aggressive≫ approach to habilitation. J Pediatr Orthop. (2007) 27:926–9. 10.1097/bpo.0b013e31815a6045 18209617

[31] Vega H Waisfisz Q Gordillo M Sakai N Yanagihara I Yamada M. Roberts syndrome is caused by mutations in ESCO2, a human homolog of yeast ECO1 that is essential for the establishment of sister chromatid cohesion. Nat Genet. (2005) 37:468–70. 10.1038/ng1548 15821733

[32] Van Maldergem L Verloes A Lejeune L Gillerot Y. The Baller-Gerold syndrome. J Med Genet. (1992) 29:266–8. 10.1136/jmg.29.4.266 1583650 PMC1015930

[33] Mégarbané A Melki I Souraty N Gerbaka J El Ghouzzi V Bonaventure J. Overlap between Baller-Gerold and Rothmund-Thomson syndrome. Clin Dysmorphol. (2000) 9:303–5. 10.1097/00019605-200009040-00018 11045594

[34] Afifi HH Abdel-Salam GMH Eid MM Tosson AMS Shousha WG Abdel Azeem AA. Expanding the mutation and clinical spectrum of Roberts syndrome. Congenit Anom. (2016) 56:154–62. 10.1111/cga.12151 26710928

[35] Mengen E Kotan LD Ucakturk SA Topaloglu AK Yuksel B. A novel frameshift mutation in ESCO2 gene in roberts syndrome. J Coll Physicians Surg Pak. (2018) 28:403–5. 10.29271/jcpsp.2018.05.403 29690975

[36] Cao DH Mu K Liu DN Sun JL Bai XZ Zhang N. Identification of novel compound heterozygous RECQL4 mutations and prenatal diagnosis of Baller-Gerold syndrome: a case report. Genet Mol Res. (2015) 14:4757–66. 10.4238/2015.May.11.8 25966250

[37] Gerkes EH van der Kevie-Kersemaekers A-MF Yakin M Smeets DFCM van Ravenswaaij-Arts CMA. The importance of chromosome studies in Roberts syndrome/SC phocomelia and other cohesinopathies. Eur J Med Genet. (2010) 53:40–4. 10.1016/j.ejmg.2009.10.005 19878742

[38] Socolov RV Andreescu NI Haliciu AM Gorduza EV Dumitrache F Balan RA. Intrapartum diagnostic of Roberts syndrome - case presentation. Rom J Morphol Embryol. (2015) 56:585–8. 26193234

[39] Temtamy SA Aglan MS Nemat A Eid M. Expanding the phenotypic spectrum of the Baller-Gerold syndrome. Genet Couns. (2003) 14:299–312. 14577674

[40] Krantz ID. Cohesin embraces new phenotypes. Nat Genet. (2014) 46:1157–8. 10.1038/ng.3123 25352100 PMC4268132

[41] Liu J Zhang Z Bando M Itoh T Deardorff MA Clark D. Transcriptional dysregulation in NIPBL and cohesin mutant human cells. PLoS Biol. (2009) 7:e1000119. 10.1371/journal.pbio.1000119 19468298 PMC2680332

[42] Larizza L Magnani I Roversi G. Rothmund-Thomson syndrome and RECQL4 defect: splitting and lumping. Cancer Lett. (2006) 232:107–20. 10.1016/j.canlet.2005.07.042 16271439

[43] Jabs EW Tuck-Muller CM Cusano R Rattner JB. Studies of mitotic and centromeric abnormalities in Roberts syndrome: implications for a defect in the mitotic mechanism. Chromosoma. (1991) 100:251–61. 10.1007/BF00344159 2055135

